# Prevalence and Clinical Characteristics of Children and Adolescents with Metabolically Healthy Obesity: Role of Insulin Sensitivity

**DOI:** 10.3390/life10080127

**Published:** 2020-07-28

**Authors:** Federica Vinciguerra, Andrea Tumminia, Roberto Baratta, Alfredo Ferro, Salvatore Alaimo, Maria Hagnäs, Marco Graziano, Riccardo Vigneri, Lucia Frittitta

**Affiliations:** 1 Endocrinology, Department of Clinical and Experimental Medicine, University of Catania, 95122 Catania, Italy; vinciguerrafederica@gmail.com (F.V.); andreatumminia82@gmail.com (A.T.); maria.hagnas@rovaniemi.fi (M.H.); graziano.marco91@gmail.com (M.G.); vigneri@unict.it (R.V.); 2 Diabetes, Obesity and Dietetic Center, Garibaldi-Nesima Medical Center, 95122 Catania, Italy; rob.baratta@gmail.com; 3 Bionformatic Unit, Department of Clinical and Experimental Medicine, University of Catania, 95125 Catania, Italy; ferro@dmi.unict.it (A.F.); alaimos@dmi.unict.it (S.A.); 4 Center for Life Course Health Research, University of Oulu, 90570 Oulu, Finland; 5 Rovaniemi Health Center, 96200 Rovaniemi, Finland; 6 Institute of Crystallography, Structural Chemistry and Biosystems, CNR-ICCSB, Catania Section, 95126 Catania, Italy

**Keywords:** metabolically healthy obesity, insulin resistance, metabolic syndrome, adolescents, children

## Abstract

Obesity represents a major risk factor for metabolic disorders, but some individuals, “metabolically healthy” (MHO), show less clinical evidence of these complications, in contrast to “metabolically unhealthy” (MUO) individuals. The aim of this cross-sectional study is to assess the prevalence of the MHO phenotype in a cohort of 246 overweight/obese Italian children and adolescents, and to evaluate their characteristics and the role of insulin resistance. Homeostasis model assessment–insulin resistance (HOMA-IR), insulin sensitivity index (ISI), insulinogenic index (IGI) and disposition index (DI) were all calculated from the Oral Glucose Tolerance Test (OGTT). MHO was defined by either: (1) HOMA-IR < 2.5 (MHO-IRes), or (2) absence of the criteria for metabolic syndrome (MHO-MetS). The MHO prevalence, according to MHO-MetS or MHO-IRes criteria, was 37.4% and 15.8%, respectively. ISI was the strongest predictor of the MHO phenotype, independently associated with both MHO-IRes and MHO-MetS. The MHO-MetS group was further subdivided into insulin sensitive or insulin resistant on the basis of HOMA-IR (either < or ≥ 2.5). Insulin sensitive MHO-MetS patients had a better metabolic profile compared to both insulin resistant MHO-MetS and MUO-MetS individuals. These data underscore the relevance of insulin sensitivity to identifying, among young individuals with overweight/obesity, the ones who have a more favorable metabolic phenotype.

## 1. Introduction

Overweight and obesity prevalence has tremendously increased in recent years, not only among adults, but also among children and adolescents [1,2]. The global prevalence of childhood overweight and obesity, calculated on the basis of body mass index (BMI, Kg/m^2^), has increased by over 50% in the last two decades (from 4.2% in 1990 to 6.7% in 2010), and it is expected to reach 9.1% by 2020 [3]. Such a secular trend has also been recently confirmed among Sicilian schoolchildren [4,5]. 

It is generally assumed that the earlier onset, and the longer duration, of obesity is associated with a greater risk of developing negative health consequences from childhood to adulthood [6], including the early occurrence of various cardio-metabolic complications, such as hypertension, impaired glucose tolerance and metabolic syndrome (MetS) [7,8].

Obesity, however, is a heterogeneous syndrome, and a benign phenotype of obesity, the so-called “metabolically healthy obesity” (MHO), has been described both in adults and younger (children) patients with obesity [9,10]. MHO patients represent a class of subjects who, despite the fat mass excess, show a favorable metabolic profile [9,11]. These individuals seem to be less prone to the traditional metabolic abnormalities that often accompany excess body fat (e.g., insulin resistance, dyslipidemia, hypertension), showing a reduced incidence of several obesity-related comorbidities [9], such as a 50% decreased risk of developing type 2 diabetes (T2D) compared with metabolically unhealthy obese (MUO) subjects [12]. Conversely, a significantly higher cardiovascular risk has been reported, compared to lean subjects [13,14,15]. 

Despite the need to identify precociously this benign metabolic condition, to date, there has been no consensus on the definition of MHO, either in adults [16] or in younger patients [11,17]. Studies investigating MHO in childhood, in fact, have used different diagnostic criteria for its definition, such as a lower level of insulin resistance [18,19,20], the absence of MetS [21,22,23,24,25], the combination of both criteria [20] or the presence of a favorable blood pressure, glucose and lipid profile, together with the absence of hepatosteatosis [26]. 

Recently, a scoping review was carried out in order to identify a shared MHO definition through consultation of experts and the application of the Delphi technique [24]. However, the consensus was not reached on all variables, and several limitations reported by the authors underlined the need for more research on the topic. 

This wide spectrum of current clinical and biochemical definitions for MHO has led to a consequently broad range of reported prevalence in young individuals. According to different authors, the MHO status among children with overweight or obesity ranges from 3% to 80% [24]. 

Notably, the concept of MHO is still controversial, due to the potentially transient nature of metabolic health status [27]. Moreover, it is unclear whether a single metabolic abnormality is the driving factor that characterizes the etiopathogenesis of this condition, or whether multiple factors contribute to the MHO phenotype.

The present study aimed at estimating the proportion of the MHO phenotype in a cohort of Italian children and adolescents with overweight or obesity, using different diagnostic criteria [19,22], and to evaluate the role of insulin resistance in determining the clinical and metabolic features of the MHO phenotype.

## 2. Methods

### 2.1. Study Procedures and Assays 

From January 2011 to June 2012, 246 children and adolescents (mean age 13 ± 3 years, range 7–18) with overweight or obesity were consecutively recruited at the Obesity and Dietetic Center of the Garibaldi-Nesima Medical Center, Catania, Sicily, Italy. Children were classified as overweight or obese based on the age- and gender-specific BMI cut-off points for children, which were developed by the International Obesity Task Force (IOTF) [28]. Overweight was defined as a BMI from the 85th to the 94th percentile, and obesity was defined as a BMI in the 95th percentile or higher [28,29]. Subjects of normal weight, those suffering from major or active concurrent illness (e.g., diabetes, congenital or acquired hypothyroidism, congenital adrenal hyperplasia, growth hormone deficiency) and/or those on regular medications (e.g., glucocorticoid, metformin, insulin) were excluded from the study. 

The physical examination was carried out by two trained investigators who measured weight and height for calculating BMI and BMI z-score [4,29], and the waist circumference (after gently exhaling, at the narrowest part between the lower rib and the iliac crest using a non-elastic flexible tape, recorded to the nearest 0.1 cm) and the waist to height ratio (WtHR) [30] were also calculated. Systolic and diastolic blood pressure (BP) was recorded before blood sampling with the subject in a sitting position and after a minimum of 5 min of acclimation. The mean of two BP measurements with 5 min interval was used in the analysis. Family and personal medical history, age of onset of obesity and the Tanner pubertal stage were recorded. According to the Tanner stage, patients were classified as pre-pubertal (stage I) and pubertal (stages II, III, IV, V) [31].

Blood samples were collected in the morning after 12 h fasting. Total and high-density lipoprotein (HDL) cholesterol (mmol/L), triglycerides, adiponectin and leptin levels were measured in the baseline sample. Plasma glucose and insulin levels were measured at baseline and 30, 60, 90 and 120 min after a 75 g oral glucose tolerance test (OGTT).

Plasma glucose was measured using the glucose oxidase method on a Beckman Glucose Analyzer 2 (Beckman Coulter, Inc., Fullerton, CA, USA), while plasma insulin was measured by the microparticle enzyme immunoassay (Abbott Laboratories, Abbott Park, IL, USA) method. Total cholesterol and triglycerides were evaluated via enzymatic methods (Instrumentation Laboratory, Milan, Italy). HDL cholesterol fraction was separated by the use of Mg^2+^ and the dextran sulfate method (Sclavo Diagnostics, Siena, Italy). Adiponectin and leptin levels were measured by RIA (Linco Research, Inc., St. Charles, MO, USA) in plasma samples, which were immediately frozen and stored at –20 °C. The homeostasis model assessment–insulin resistance (HOMA-IR) was calculated according to the Equation (1):

fasting insulin (mU/L) × fasting glucose (mmol/L)/22.5(1)

Early-phase insulin secretion (insulinogenic index, IGI) was calculated according to the Equation (2):

[insulin (mU/L) at 30 min–fasting plasma insulin (mU/L)]/[glucose (mmol/L) at 30 min–fasting plasma glucose (mmol/L)](2)

Insulin sensitivity (insulin sensitivity index, ISI) was calculated according to the following Equation (3):

10,000/√[fasting plasma glucose (mg/dL) × fasting plasma insulin (mU/L)] × [mean OGTT glucose concentration (mg/dL) × mean OGTT insulin concentration (mU/L)](3)

The disposition index (DI) was the product of IGI and ISI [32,33].

As already mentioned, numerous different criteria have been proposed for defining MHO. Table 1 summarizes the different criteria that have until now been used for the definition of the MHO status in children and adolescents.

In order to capture the most important metabolic characteristics of MHO individuals, we chose to use two different criteria to define the presence of MHO in our study [19,22]. Subjects were considered (A) “insulin sensitive” (MHO-IRes) or “insulin resistant” (MUO-IRes) if HOMA-IR was either < or ≥ 2.5, respectively [34,35,36], and (B) metabolically healthy (MHO-MetS) or unhealthy (MUO-MetS) in the absence or the presence of at least one of the criteria indicated by the National Cholesterol Education Program’s Adult Treatment Panel (NCEP-ATP) for the MetS (adapted for children and adolescents) [34] (i.e., fasting plasma glucose ≥ 100 mg/dL, triglycerides ≥ 95th percentile, HDL cholesterol ≤ 5th percentile, systolic or diastolic blood pressure ≥ 95th percentile [20,37]). In addition, the MHO-MetS individuals were further subdivided into insulin sensitive MHO-MetS or insulin resistant MHO-MetS, according to the HOMA index being either < or ≥ 2.5, respectively (Table 2).

The study was conducted in accordance with the Declaration of Helsinki and its later amendments. The protocol was approved by the Ethics Committee of Garibaldi Hospital, Catania 2 (949/CE), and a written informed consent was obtained from the parents of all examined subjects before the commencement of the study.

### 2.2. Statistical Analysis

Data are expressed as mean ± standard deviations (SD) for continuous variables, and number of cases and percentage (%) for categorical variables. Continuous variables were compared between groups using either the unpaired t test, the one-way ANOVA or nonparametric analysis of covariance (ANCOVA), where appropriate. Differences in categorical variables were determined by Pearson’s χhi-squared test. A multivariate logistic regression model accounting for possible confounders (BMI z-score, WC, WtHR, SBP, DBP, HDL cholesterol, triglycerides, fasting plasma glucose) was applied to examine the associations between each biochemical and clinical parameter and the MHO status. A Hosmer–Lemeshow post estimation test was used to assess the model’s performance. The odds ratios’ (OR) confidence interval (CI) was calculated at 95%. A *p* value < 0.05 was considered to be statistically significant.

Data were analyzed using StatView 5.01 (SAS Institute, Cary, NC, USA). Calculation of frequencies was carried out using the STATA 14.2 SE software (STATA Corp., College Station, TX, USA).

## 3. Results

The prevalence of MHO was clearly different according to the criteria used; lower for MHO-IRes (39 cases, 15.8%) and higher for MHO-MetS (92 cases, 37.4%).

The clinical, biochemical and anthropometric characteristics of the studied subjects are summarized in Table 3. Regardless of the definition used, both MHO-IRes and MHO-MetS patients showed a favorable metabolic profile compared to MUO; lower BMI z-score, WC and WtHR (although not statistically significant for MHO-MetS), lower mean systolic and diastolic blood pressure, lower fasting plasma insulin, glycaemia and triglycerides (although not statistically significant for MHO-IRes), as well as higher HDL cholesterol.

No difference was observed between the MHO and MUO groups regarding age, gender, being pre-pubertal/pubertal, pubertal stage, and mean age at the onset of obesity.

### 3.1. Adipokines: Leptin and Adiponectin

Low levels of leptin were found in MHO individuals, however they were classified; leptin values were significantly lower in MHO-MetS compared to MUO-MetS (34.0 ± 19.0 vs. 27.9 ± 21.4 ng/mL; *p* = 0.01), and in MHO-IRes compared to MUO-IRes (20.1 ± 13.9 vs. 33.9 ± 20.4 ng/mL; *p* < 0.01). Conversely, adiponectin levels were not significantly different between the two groups (Table 3).

### 3.2. OGTT-Patterns and Derived Indices

Significantly lower values in the HOMA index were observed in MHO individuals, however they were classified (Table 3). ISI, a more valuable index of insulin sensitivity derived from OGTT, was significantly higher in both MHO-IRes (4.9 ± 1.5 vs. 2.4 ± 1.0, *p* < 0.01) and MHO-MetS (3.2 ± 1.5 vs. 2.5 ± 1.3; *p* < 0.01) patients, compared to their MUO counterparts. Moreover, MHO-IRes patients showed a significantly lower IGI (30.3 ± 17.9 vs. 49.0 ± 25.7, *p* < 0.01) and a significantly higher DI (139.9 ± 81.0 vs. 110.0 ± 68.1, *p* = 0.01) compared to MUO-IRes, suggesting their lower risk of developing T2D later in life [33].

### 3.3. Multivariate Logistic Regression Analyses

Multivariate logistic regression analyses, accounting for the possible confounders related to the two definitions used for MHO (BMI z-score, WC, SBP, DBP, HDL cholesterol, triglycerides, fasting plasma glucose), showed that ISI was the strongest predictor of the MHO phenotype. In fact, it was the only parameter independently associated with MHO status regardless of the definition used; the OR was 7.49 (95% CI 3.80–14.84; *p* < 0.01) for MHO-IRes and 1.38 (95% CI 1.09–1.74; *p* < 0.01) for MHO-MetS (Table 4).

On the contrary, as expected on the basis of the criteria used for the MetS definition, SBP, DBP and fasting plasma glucose were significantly negatively associated with MHO-MetS, whereas HDL cholesterol was positively associated. Finally, leptin levels were independently and negatively associated with MHO-IRes (OR 0.96, 95% CI 0.92–0.99, *p* = 0.04) (Table 4).

### 3.4. MHO-MetS Subgroups According to Insulin Sensitivity

**Figure 1:**
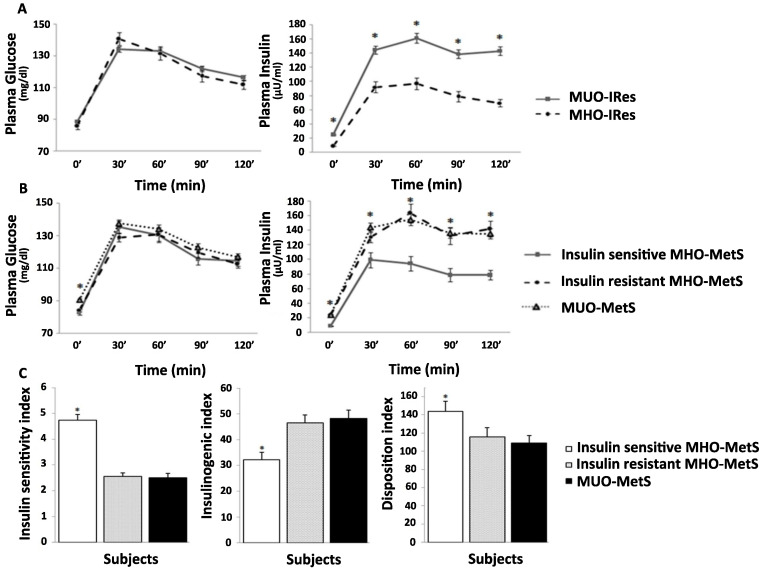
Plasma glucose and insulin levels during OGTT in MHO-IRes vs. MUO-IRes (**A**). Plasma glucose and insulin levels (**B**) and OGTT-derived indices (**C**) in insulin sensitive MHO-MetS, insulin resistant MHO-MetS and MUO-MetS subjects. * Indicates significant differences.

In order to deeply evaluate the impact of insulin sensitivity on MHO, we further subdivided patients with MHO-MetS into two groups: the insulin sensitive and the insulin resistant ones, on the basis of the HOMA-IR < or ≥ 2.5. Insulin sensitive MHO-MetS patients showed a more favorable clinical and metabolic phenotype, not only when compared to MUO-MetS, but also in comparison with insulin resistant MHO-MetS (lower BMI z-score, WC, WtHR and leptin levels) (Table 5). This better metabolic condition is also noticeable when comparing insulin sensitive MHO-MetS OGTT-patterns and derived indices with those of insulin resistant MHO-MetS and MUO-MetS. Insulin sensitive MHO-MetS patients, in fact, exhibited lower fasting and post-load plasma insulin levels (Figure 1B), higher ISI and DI, and lower IGI (Figure 1C) compared to both insulin resistant MHO-MetS and MUO-MetS, and exhibited an insulin profile similar to those with MHO-IRes (Figure 1A,B).

**Figure 2:**
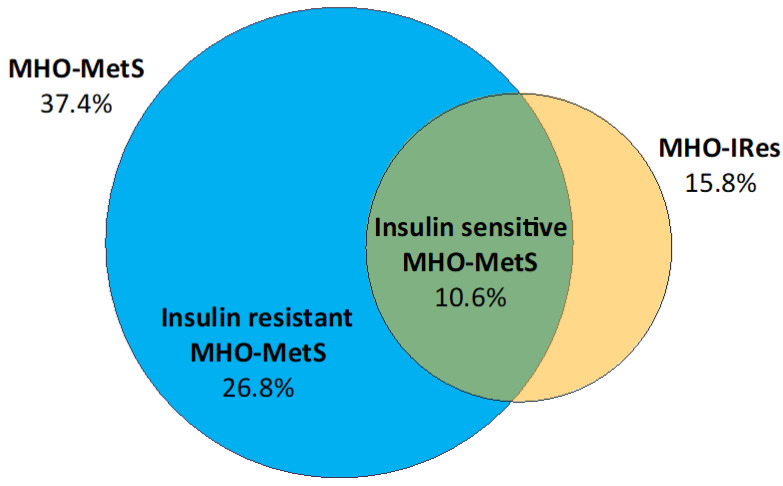
Prevalence of the MHO condition. In our cohort of subjects, 37.4% had none of the criteria of metabolic syndrome (MHO-MetS), and 15.8% were classified as MHO on the basis of the absence of insulin resistance (HOMA-IR < 2.5, MHO-IRes). The blue area of the Venn diagram represents MHO-MetS having HOMA-IR ≥ 2.5 (insulin resistant MHO-MetS). The overlap of the two definitions (the green area) identifies the sub-cohort of MHO-MetS with HOMA-IR < 2.5 (insulin sensitive MHO-MetS).

According to these three different definitions used, we then evaluated the prevalence of MHO in our population. Using HOMA-IR in addition to the MetS criteria allows us to identify a cluster of patients (10.6%) that really do not have any metabolic abnormality (Figure 2).

## 4. Discussion

The first aim of the present study was to determine the prevalence of MHO in our cohort of children and adolescents using two currently applied criteria for the diagnosis of MHO [19,22]. The first is based on the HOMA-IR value (MHO-IRes) and the second is based on the MetS criteria (MHO-MetS). Our data showed that the prevalence of MHO in children with overweight or obesity was substantially different depending on the criteria used (15.8% vs. 37.4%, respectively). The frequency of MHO-IRes in our cohort of patients is similar to that previously reported in adult individuals, using the same HOMA-IR cut-off value (<2.5) [34,35,36]. Considering the possible development of transient insulin resistance during puberty, other authors used a higher HOMA-IR cut-off (<3.16) in adolescents and observed a higher prevalence of MHO [19]. Indeed, using the same HOMA-IR cut-off value, we would have observed a similarly high prevalence of MHO (25.2%). This percentage, however, remains noticeably lower compared to that of MHO-MetS, confirming the need to assess the level of insulin sensitivity in order to adequately characterize the MHO status. To point out the role of puberty in the MHO/MUO status, we calculated the percentages of each Tanner stage in our cohort, without finding any significant difference.

Using the NCEP-ATP criteria for MetS, modified for percentiles cut-offs in children and adolescents [37], we found a similar prevalence of MHO-MetS compared to recent studies in adolescents [22].

Regardless of the criteria used for identifying the MHO condition, MUO individuals always had an unfavorable metabolic profile compared to MHO (higher BMI and visceral adiposity, and worse glycemic, lipid and blood pressure profile). Furthermore, serum leptin levels were also significantly higher in MUO compared to MHO, confirming the role of this adipokine in determining insulin resistance and metabolic impairment independently of the age and BMI-z score of the population studied [38]. In our series, adiponectin levels did not differ significantly between MUO and MHO individuals, at variance with the positive correlation reported by others between serum adiponectin concentrations and the MHO phenotype in children and adolescents with overweight or obesity [39,40]. This difference might be due to different ethnicities or to the assay utilized for the determination of adiponectin (radioimmunoassay—RIA vs. enzyme-linked immunosorbent assay—ELISA).

The second aim of the present study was to identify the role of insulin resistance in determining clinical and metabolic features of the MHO phenotype in young individuals. We found that ISI was the strongest predictor of MHO, being independently associated with both MHO-IRes and MHO-MetS. This observation supports the crucial role of insulin sensitivity in the pathophysiology of MHO. As already demonstrated by “clamp-based” studies, ISI calculated on the basis of post-load plasma glucose and insulin levels provides a good approximation of both hepatic and skeletal muscle disposition of glucose, and correlates well with insulin sensitivity measured directly using the euglycemic-hyperinsulinemic clamp [41,42]. ISI, however, cannot be used for routine clinical practice, because it requires an OGTT, and therefore cannot be proposed on a large-scale basis. Nevertheless, insulin resistance can be more easily assessed by calculating HOMA-IR, which in our study was negatively associated with MHO regardless of the criteria used.

Given this relevant role of insulin sensitivity in MHO, we further subdivided our young MetS patients into either insulin sensitive MHO-MetS or insulin resistant MHO-MetS. Considering the degree of insulin resistance, MHO-MetS patients that were insulin sensitive had a significantly better metabolic profile, not only compared to MUO, but also compared to insulin resistant MHO-MetS (e.g., patients classified as “healthy” according to MetS criteria). These findings suggest that using the MetS classification in identifying MHO patients can overestimate the prevalence of MHO phenotypes in those young subjects with overweight/obesity whose condition is predominantly characterized by impaired insulin sensitivity. Insulin resistance represents one of the main etiological features leading to metabolic impairment, irrespective of the excess of adipose tissue [43,44]. This condition, in fact, although strongly related to obesity, could affect also normal weight young individuals [45,46,47,48]. Moreover, insulin resistance per se is an independent predictor of cardiovascular risk both in childhood [48] and in adulthood [49], strongly related to atherogenic dyslipidemia (low HDL cholesterol and hypertriglyceridemia) and hypertension, and deeply involved in the pathogenesis of T2D, representing the “trigger” of MetS. For these reasons, the presence of insulin resistance, along with the absence of other components of MetS in childhood, should be considered a risk factor for metabolic disorders [50] and cardiovascular diseases [51]. It has been demonstrated that insulin resistance affects myocardial function [52,53,54]. Recently, Corica et al. showed the negative effects of insulin resistance on cardiovascular remodeling and subclinical myocardial dysfunction in a cohort of children with obesity, and suggested that insulin resistance represents a strong predictor of subclinical myocardial dysfunction in obese, non-diabetic children [55].

Therefore, in light of this evidence, the definition of MHO must also take into account the presence of insulin resistance.

Another significant finding is that in insulin sensitive MHO-MetS patients, DI is higher compared to both MUO and insulin resistant MHO-MetS individuals. This index reflects the ability of the pancreatic β-cells to compensate for insulin resistance [56]. A low DI, in fact, is considered an early marker of insufficient β-cell compensation, correlated, therefore, with the risk of future diabetes [57]. The decreased DI, observed in both MUO-MetS and insulin resistant MHO-MetS patients, suggests therefore that these young individuals do not only presently have an unfavorable metabolic profile, but also a higher risk of developing T2D in the future.

## 5. Conclusions

Despite several limitations, such as the lack of information regarding long-term outcomes (e.g., obesity complications and related morbidities) due to the cross-sectional design of our study, our study offers important insights into the MHO phenotype during childhood and adolescence. Clinicians should consider the heterogeneity of pediatric obesity and the crucial role of insulin sensitivity in determining MHO. In particular, we believe that, in addition to the absence of the metabolic syndrome criteria, the degree of insulin sensitivity should be assessed in overweight/obese children and adolescents in order to identify those at low risk. We demonstrated, in fact, that if we exclusively used the metabolic syndrome criteria for the MHO definition, there would have been an overestimation of the number of patients without metabolic alterations. We propose, therefore, to use both the described criteria in order to diagnose as MHO only those individuals at really low metabolic risk.

The possibility to accurately distinguish young individuals with overweight/obesity by assessing insulin sensitivity could provide better and more appropriate therapeutic options, with a different intensity of intervention according to the risk level, and more structured interventions (e.g., multidisciplinary obesity management and/or bariatric surgery) in individuals at higher risk.

Further longitudinal studies are needed so as to better characterize the evolution and outcomes of MHO young patients.

## Figures and Tables

**Table 1:** Criteria used for the definition of Metabolically Healthy Obesity (MHO) in children and its prevalence across studies.

Method Used to Identify MHO Individuals	Reference	Years	Country	MHO Prevalence
• ≤1 of the following: TG ≥ 110 mg/dL, HDL-C < 40 mg/dL, SBP/DBP ≥ 90th percentile for age and gender and height, and FPG ≥ 100 mg/dL	[23]	2013	USA	68%
• Homeostasis model assessment–insulin resistance (HOMA-IR) ≤ 2.75	[18]	2013	Serbia	25%
• Absence of age- and gender-specific cut-off points for FG, TG, HDL- C, SBP and DBP	[26]	2013	Canada	25%
• Absence of: TG ≥ 150 mg/dL, HDL-C < 40 mg/dL (males) and <50 (females), SBP ≥ 130 mmHg or DBP ≥ 85 mmHg, FPG ≥ 100 mg/dL	[21]	2013	Austria	16%
• A: HOMA-IR < 3.16 • B: Absence of: SBP or DBP ≥ 90th percentile for age, gender and height; TG ≥ 25 mmol/L; HDL-C ≥ 1.02 mmol/L; FPG ≥ 5.6 mmol/L. • C: A + B	[19]	2014	Canada	A: 21.5% B: 31.5% C: 11.6%
• A: Absence of: HDL-C < 40 mg/dL (males) and <50 mg/dL (females), TG ≥ 150 mg/dL; SBP ≥ 130 mmHg or DBP ≥ 85 mmHg; FPG ≥ 100 mg/dL • B: HOMA-IR < 3.16 • C: A + B	[20]	2016	Belgium	A: 18.6% B: 19.2% C: 6.4%
• A: HOMA-IR ≤ 2.3 • B: Absence of: SBP or DBP ≥ 90th percentile for age, gender and height, TG ≥ 1.24 mmol/L; HDL-C ≤ 1.03 mmol/L, FPG ≥ 5.6 mmol/L	[22]	2016	China	A: 27.1% B: 37.2%
• Absence of sex- and age-specific metabolic syndrome cut-off points for FG, TG, HDL- C, SBP and DBP	[25]	2017	Europe	80%
• A: Absence of: SBP or DBP ≥ 90th percentile for age, gender and height, TG ≥ 110 mg/dL, HDL-C < 40 mg/dL and FPG ≥ 110 mg/dL • B: Absence of: elevated SBP or DBP (≥120/80 mmHg), TG ≥ 150 mg/dL, HDL-C < 40 mg/dL, FPG ≥ 100 mg/dL	[14]	2019	Brazil, China, Greece, Italy Spain	A: 4.5% B: 8.2%
•A: HOMA-IR < 2.5 (MHO-IRes) • B: Absence of: FPG ≥ 100 mg/dL, TG ≥ 95th percentile, HDL-C ≤ 5th percentile, SBP or DBP ≥ 95th percentile (MHO-MetS) • C: A + B (insulin sensitive MHO-MetS)	Present study	2020	Italy	A: 15.8% B: 37.4% C: 10.6%

Abbreviations: FPG, fasting plasma glucose; SBP, systolic blood pressure; DBP, diastolic blood pressure: TG, triglycerides; HDL-C, HDL cholesterol.

**Table 2:** Diagnostic criteria for MHO. All criteria must be fulfilled.

MHO-IRes	MHO-MetS	Insulin Sensitive MHO-MetS	Insulin Resistant MHO-MetS
HOMA index < 2.5	FPG < 100 mg/dL	FPG < 100 mg/dL	FPG < 100 mg/dL
	TG ≤ 95th percentile	TG ≤ 95th percentile	TG ≤ 95th percentile
	HDL-C ≥ 5th percentile	HDL-C ≥ 5th percentile	HDL-C ≥ 5th percentile
	SBP or DBP ≤ 95th percentile	SBP or DBP ≤ 95th percentile	SBP or DBP ≤ 95th percentile
		HOMA index < 2.5	HOMA index ≥ 2.5

Abbreviations: MHO-MetS, metabolically healthy obese/overweight children/adolescents according to the absence of the criteria for metabolic syndrome; MHO-IRes, metabolically healthy obese/overweight children/adolescents according to HOMA-IR < 2.5; FPG, fasting plasma glucose; HDL-C, HDL cholesterol; TG, triglycerides; SBP, systolic blood pressure; DBP, diastolic blood pressure.

**Table 3:** Anthropometric and biochemical characteristics of overall patients, according to the two diagnostic criteria for MHO.

Characteristics	Overall n = 246	MUO-IRes n = 207	MHO-IRes n = 39	*p*	MUO-MetS n = 154	MHO-MetS n = 92	*p*
Age (years)	12.8 ± 2.8	12.8 ± 2.8	12.9 ± 3.1	0.71	12.8 ± 2.7	12.8 ± 3.1	0.89
Males (n, %)	126 (51.2)	102 (49.3)	25 (64.1)	0.08	80 (51.9)	46 (50)	0.77
Pubertal–Tanner stages 2–5 (n, %)	127 (51.6)	111 (53.6)	16 (41.0)	0.20	80 (51.9)	47 (51.1)	0.75
Tanner stage 3 (n, %)	30 (12.2)	26 (12.6)	4 (10.2)	0.69	21 (13.6)	9 (9.8)	0.37
Tanner stage 5 (n, %)	70 (28.4)	59 (28.5)	11 (28.2)	0.87	41 (26.6)	29 (31.5)	0.41
Early obesity onset (<5 years) (n, %)	126 (51.2)	103 (49.8)	22 (56.4)	0.67	80 (51.9)	46 (50)	0.94
BMI z-score	2.5 ± 0.5	2.6 ± 0.5	2.2 ± 0.3	**<0.01**	2.5 ± 0.5	2.4 ± 0.5	0.09
WC (cm)	106.1 ± 13.6	107.5 ± 13.4	97.9 ± 11.3	**<0.01**	107.1 ± 13.8	104.3 ± 13.0	0.06
WtHR	0.68 ± 0.07	0.68 ± 0.07	0.64 ± 0.05	**<0.01**	0.68 ± 0.07	0.67 ± 0.06	0.16
SBP (mmHg)	111.6 ± 13.1	112.3 ± 13.4	107.8 ± 10.5	**0.02**	115.1 ± 13.5	105.9 ± 10.0	**<0.01**
DBP (mmHg)	69.7 ± 8.8	70.1 ± 9.1	67.2 ± 7.1	0.05	72.3 ± 9.0	65.3 ± 6.5	**<0.01**
Total cholesterol (mg/dL)	156.7 ± 28.5	156.0 ± 28.9	159.9 ± 26.0	0.44	157.5 ± 30.9	155.2 ± 23.9	0.52
HDL-C (mg/dL)	40.0 ± 10.0	39.5 ± 10.0	43.3 ± 8.8	**0.01**	36.7 ± 10.7	42.2 ± 8.0	**<0.01**
Triglycerides (mg/dL)	92.3 ± 45.8	94.5 ± 46.8	80.5 ± 38.0	0.06	97.8 ± 49.6	83.1 ± 36.8	**0.01**
LDL-C (mg/dL)	98.2 ± 24.5	97.7 ± 24.8	100.5 ± 22.6	0.46	99.3 ± 26.3	96.3 ± 20.9	0.34
FPG (mg/dL)	88.1 ± 11.9	88.5 ± 12.0	85.7 ± 11.5	0.18	90.5 ± 13.1	83.9 ± 8.0	**<0.01**
Fasting plasma insulin (µU/mL)	22.3 ± 10.9	24.9 ± 10.0	8.9 ± 2.2	**<0.01**	24.1 ± 10.8	19.4 ± 10.4	**<0.01**
2 h OGTT plasma glucose (mg/dL)	115.6 ± 21.5	116.3 ± 22.3	111.8 ± 16.1	0.23	116.9 ± 22.7	113.2 ± 18.8	0.20
2 h OGTT plasma insulin (µU/mL)	131.2 ± 85.0	142.7 ± 86.8	69.5 ± 33.1	**<0.01**	135.2 ± 86.3	124.3 ± 82.6	0.30
IFG, IGT, T2D (n, %)	58 (23.6)	51 (24.6)	7 (17.9)	0.37	50 (32.5)	8 (8.7)	**<0.01**
HOMA-IR	4.9 ± 2.4	5.4 ± 2.3	1.9 ± 0.5	**<0.01**	5.3 ± 2.5	4.1 ± 2.3	**<0.01**
ISI	2.8 ± 1.4	2.4 ± 1.0	4.9 ± 1.5	**<0.01**	2.5 ± 1.3	3.2 ± 1.5	**<0.01**
IGI	46.1 ± 25.5	49.0 ± 25.7	30.3 ± 17.9	**<0.01**	48.2 ± 26.6	42.5 ± 23.5	0.09
DI	114.8 ± 70.9	110.0 ± 68.1	139.9 ± 81.0	**0.01**	109.2 ± 67.6	124.0 ± 75.6	0.11
Leptin (ng/mL)	31.8 ± 21.2	33.9 ± 20.4	20.1 ± 13.9	**<0.01**	34.0 ± 19.0	27.9 ± 21.4	**0.01**
Adiponectin (µg/mL)	12.2 ± 5.6	12.0 ± 5.5	13.4 ± 5.7	0.14	12.1 ± 5.5	12.4 ± 5.7	0.61

Abbreviations: BMI, body mass index; WC, waist circumference; WtHR, waist to height ratio; SBP, systolic blood pressure; DBP, diastolic blood pressure; HDL-C, HDL cholesterol; LDL-C, LDL cholesterol; FPG, fasting plasma glucose; OGTT, oral glucose tolerance test; IFG, impaired fasting glucose; IGT, impaired glucose tolerance; T2D, type 2 diabetes; HOMA-IR, Homeostasis model assessment–insulin resistance; ISI, insulin sensitivity index; IGI, insulinogenic index; DI, disposition Index. Bold text indicates a statistically significant difference (*p* value < 0.05).

**Table 4:** Multivariate logistic regression analyses between anthropometric and metabolic parameters and the MHO condition, defined according to the two different criteria (MHO-IRes, MHO-MetS).

	MHO-IRes		MHO-MetS	
	OR (95% CI)	*p*	OR (95% CI)	*p*
BMI z-score	0.15 (0.02–1.10)	0.06	0.65 (0.21–2.00)	0.45
WC	1.04 (0.96–1.11)	0.31	0.99 (0.96–1.02)	0.66
SBP	1.02 (0.96–1.08)	0.64	0.96 (0.93–0.99)	**0.03**
DBP	0.96 (0.86–1.05)	0.36	0.85 (0.81–0.90)	**<0.01**
HDL-C	1.02 (0.96–1.08)	0.56	1.07 (1.03–1.12)	**<0.01**
Triglycerides	1.00 (0.99–1.02)	0.40	0.99 (0.98–1.01)	0.47
FPG	0.99 (0.94–1.05)	0.85	0.88 (0.85–0.92)	**<0.01**
ISI	7.49 (3.80–14.84)	**<0.01**	1.38 (1.09–1.74)	**<0.01**
IGI	0.98 (0.95–1.01)	0.25	0.99 (0.98–1.01)	0.38
DI	0.99 (0.98–1.01)	0.20	1.00 (0.99–1.01)	0.65
Leptin	0.96 (0.92–0.99)	**0.04**	0.98 (0.97–1.01)	0.26
Adiponectin	0.96 (0.87–1.05)	0.39	0.99 (0.93–1.06)	0.81

Multivariate logistic regression analyses were corrected for BMI z-score, WC, SBP, DBP, HDL-C, Triglycerides and FPG. Abbreviations: BMI, body mass index; WC, waist circumference; SBP, systolic blood pressure; DBP, diastolic blood pressure; HDL-C, HDL cholesterol; LDL-C, LDL cholesterol; FPG, fasting plasma glucose; ISI, insulin sensitivity index; IGI, insulinogenic index; DI, disposition Index. Bold text indicates a statistically significant difference (*p* value < 0.05).

**Table 5:** Anthropometric and biochemical characteristics of MUO-MetS patients compared to both insulin resistant MHO-MetS and insulin sensitive MHO-MetS.

	MUO-MetS n = 154	MHO-MetS n = 92		*p*
		Insulin Resistant MHO-MetS n = 66	Insulin Sensitive MHO-MetS n = 26	
Age (yrs)	12.8 ± 2.7	12.8 ± 2.9	12.9 ± 3.4	0.81
Males (n, %)	80 (51.9)	30 (45.4)	16 (61.5)	0.30
Pubertal–Tanner stages 2–5 (n, %)	80 (51.9)	36 (54.5)	14 (53.8)	0.80
Tanner stage 3 (n, %)	21 (13.6)	6 (9.1)	3 (11.5)	0.72
Tanner stage 5 (n, %)	41 (26.6)	22 (33.3)	7 (26.9)	0.55
Early obesity onset (<5 years) (n, %)	80 (51.9)	30 (45.4)	16 (61.5)	0.80
BMI z-score	2.5 ± 0.5	2.5 ± 0.4	2.2 ± 0.3 *°	**0.02**
WC (cm)	107.1 ± 13.8	106.4 ± 12.8	99.0 ± 12.2 *°	**0.01**
WtHR	0.68 ± 0.07	0.68 ± 0.06	0.65 ± 0.05 *°	**0.01**
SBP (mmHg)	115.1 ± 13.5	105.4 ± 10.4 *	107.0 ± 8.7 *	**<0.01**
DBP (mmHg)	72.3 ± 9.0	64.8 ± 6.8 *	66.4 ± 5.7 *	**<0.01**
Total cholesterol (mg/dL)	157.5 ± 30.9	153.6 ± 21.8	159.1 ± 28.6	0.56
HDL-C (mg/dL)	36.7 ± 10.7	41.8 ± 8.5 *	43.4 ± 6.7 *	**0.01**
Triglycerides (mg/dL)	97.8 ± 49.6	85.1 ± 36.3	78.2 ± 38.6 *	**0.04**
LDL-C (mg/dL)	99.3 ± 26.3	94.8 ± 19.9	100.1 ± 23.3	0.42
FPG (mg/dL)	90.5 ± 13.1	84.4 ± 8.0 *	82.8 ± 8.0 *	**<0.01**
Fasting plasma insulin (µU/mL)	24.1 ± 10.8	23.6 ± 9.2	8.7 ± 2.4 *°	**<0.01**
2 h OGTT plasma glucose (mg/dL)	116.9 ± 22.7	112.7 ± 19.7	114.5 ± 16.6	0.32
2 h OGTT plasma insulin (µU/mL)	135.2 ± 86.3	141.5 ± 89.1	78.6 ± 32.7 *°	**<0.01**
IFG, IGT, T2D (n, %)	50 (32.5)	7 (10.6) *	1 (3.8) *	**<0.01**
HOMA-IR	5.3 ± 2.5	4.9 ± 2.1	1.8 ± 0.5 *°	**<0.01**
ISI	2.5 ± 1.3	2.6 ± 1.0	4.7 ± 1.2 *°	**<0.01**
IGI	48.2 ± 26.6	46.5 ± 25.0	32.2 ± 14.8 *°	**<0.01**
DI	109.2 ± 67.6	116.1 ± 80.8	143.8 ± 56.7 *	**0.02**
Leptin (ng/mL)	34.0 ± 19.0	31.9 ± 22.3	17.9 ± 15.5 *°	**<0.01**
Adiponectin (µg/mL)	12.1 ± 5.5	12.0 ± 5.5	13.4 ± 6.0	0.38

* Statistically significant difference vs. MUO-MetS; ° Statistically significant difference vs. insulin resistant MHO-MetS. Abbreviations: BMI, body mass index; WC, waist circumference; WtHR, waist to height ratio; SBP, systolic blood pressure; DBP, diastolic blood pressure; HDL-C, HDL cholesterol; LDL-C, LDL cholesterol; FPG, fasting plasma glucose; OGTT, oral glucose tolerance test; IFG, impaired fasting glucose; IGT, impaired glucose tolerance; T2D, type 2 diabetes; HOMA-IR, Homeostasis model assessment–insulin resistance; ISI, insulin sensitivity index; IGI, insulinogenic index; DI, disposition Index. Bold text indicates a statistically significant difference (*p* value < 0.05).
